# Risk factors for CKD progression in Japanese patients: findings from the Chronic Kidney Disease Japan Cohort (CKD-JAC) study

**DOI:** 10.1007/s10157-016-1309-1

**Published:** 2016-07-13

**Authors:** Daijo Inaguma, Enyu Imai, Ayano Takeuchi, Yasuo Ohashi, Tsuyoshi Watanabe, Kosaku Nitta, Tadao Akizawa, Seiichi Matsuo, Hirofumi Makino, Akira Hishida, For The Chronic Kidney Disease Japan Cohort Study Group

**Affiliations:** 1 Department of Nephrology, Fujita Health University School of Medicine, 1-98, Dengakugakubo, Kutsukake-cho, Toyoake, Aichi 470-1192 Japan; 2 Nakayamadera Imai Clinic, Takarazuka, Hyogo Japan; 3 Keio University, Shinjuku-ku, Tokyo, Japan; 4 Chuo University, Bunkyo-ku, Tokyo, Japan; 5 Fukushima Medical University, Fukushima, Fukushima Japan; 6 Tokyo Women’s Medical University, Shinjuku-ku, Tokyo, Japan; 7 Showa University, Shinagawa-ku, Tokyo, Japan; 8 Nagoya University, Nagoya, Aichi Japan; 9 Okayama University, Okayama, Okayama Japan; 10 Yaizu City Hospital, Yaizu, Shizuoka Japan

**Keywords:** Chronic kidney disease, Estimated glomerular filtration rate, Urine albumin-to-creatinine ratio

## Abstract

**Background:**

Chronic kidney disease (CKD) eventually progresses to end-stage renal disease (ESRD). However, risk factors associated with CKD progression have not been well characterized in Japanese patients with CKD who are less affected with coronary disease than Westerners.

**Methods:**

A large-scale, multicenter, prospective, cohort study was conducted in patients with CKD and under nephrology care, who met the eligibility criteria [Japanese; age 20–75 years; and estimated glomerular filtration rate (eGFR): 10–59 mL/min/1.73 m^2^]. The primary endpoint was a composite of time to a 50 % decline in eGFR from baseline or time to the initiation of renal replacement therapy (RRT). The secondary endpoints were the rate of decline in eGFR from baseline, time to a 50 % decline in eGFR from baseline, time to the initiation of RRT, and time to doubling of serum creatinine (Cre) concentration.

**Results:**

2966 patients (female, 38.9 %; age, 60. 3 ± 11.6 years) were enrolled. The incidence of the primary endpoint increased significantly (*P* < 0.0001) in concert with CKD stage at baseline. The multivariate Cox proportional hazards models revealed that elevated systolic blood pressure (SBP) [hazard ratio (HR) 1.203, 95 % confidence interval (CI) 1.099–1.318)] and increased albumin-to-creatinine ratio (UACR ≥ 1000 mg/g Cre; HR: 4.523; 95 % CI 3.098–6.604) at baseline were significantly associated (*P* < 0.0001, respectively) with the primary endpoint.

**Conclusions:**

Elevated SBP and increased UACR were risk factors that were significantly associated with CKD progression to ESRD in Japanese patients under nephrology care.

UMIN clinical trial registry number: UMIN000020038.

**Electronic supplementary material:**

The online version of this article (doi:10.1007/s10157-016-1309-1) contains supplementary material, which is available to authorized users.

## Introduction

Chronic kidney disease (CKD) eventually progresses to end-stage renal disease (ESRD: maintenance dialysis or kidney transplantation). The proportion of patients with CKD is higher in Japan than in foreign countries. The estimated number of CKD patients is approximately 13.3 million, representing about 13 % of the Japanese adult population [1]. Risk factors—which are associated with the declined renal function of patients with CKD—include hypertension, diabetes mellitus (DM), cardiovascular disease (CVD), proteinuria, anemia, and administration of angiotensin receptor blockers (ARBs) [2].

The Chronic Kidney Disease Japan Cohort (CKD-JAC) study was planned in consideration of the Chronic Renal Insufficiency Cohort (CRIC) study in the US [3, 4]. The CKD-JAC Study is uniquely featured by the facts that its cohort consisted of a single race and that all enrolled patients were treated by nephrologists. Racial differences in CKD progression to ESRD have been noted between blacks and whites [5]. The rate of decline in estimated glomerular filtration rate (eGFR) is faster in Asians than in Caucasians [6]; however, the Kidney Disease: improving Global Outcomes CKD Work Group 2012 clinical practice guideline [7] does not contain the description. Nevertheless, any well-designed prospective clinical study has not been conducted to examine the renal prognosis of Japanese patients with CKD who were under nephrology care. The objective of the present study was to identify risk factors for CKD progression to ESRD in these patients.

## Subjects and methods

### Study organization

The CKD-JAC study established the Steering Committee, the Event Assessment Panel, as well as the Data Coordinating Center and determined 17 participating medical institutions, the central laboratories, and scientific advisors. The study protocol was approved by the institutional review boards at the institutions, and the study was conducted in accordance with the Declaration of Helsinki of 1975, as revised in 2005, and in compliance with the current regulations.

### Study design

The CKD-JAC study was designed as a large-scale, multicenter, prospective, cohort study (UMIN clinical trial registry number: UMIN000020038). The design and cohort thereof have been reported previously [3]. Recruited patients were screened based on eGFR, were enrolled after checking according to the eligibility and exclusion criteria, and underwent physical examination and laboratory tests (e.g., hematology, blood biochemistry, anthropometric measurements, and urinalysis).

### Study population

The CKD-JAC Study was conducted between April 2007 and March 2013 in Japanese patients with CKD who were under nephrology care. The study recruited 3087 patients at 17 medical institutions, from whom 121 were withdrawn, excluded, or lost to follow-up. The key eligibility criteria were Japanese, age 20–75 years, eGFR: 10–59 mL/min/1.73 m^2^, no initiation of renal replacement therapy (RRT), and provision of written informed consent. The key exclusion criteria were polycystic kidney disease (PKD), human immunodeficiency virus infection, cirrhosis, cancer bearing, cancer treatment in the past 2 years, and renal transplantation. Furthermore, patients were excluded from statistical analyses when retracting previous consent completely or having no information at 6 months after the study onset. All patients gave written informed consent before enrollment in the study.

### Primary endpoint

The primary endpoint was defined as a composite of two renal events: time to a 50 % decline in eGFR from baseline or time to the initiation of RRT. The following formulae for Japanese individuals were used to calculate eGFR by gender [8]: for males, eGFR (mL/min/1.73 m^2^) = 194 × [age]^−0.287^ × [serum creatinine (mg/dL)]^−1.094^; and for females, eGFR (mL/min/1.73 m^2^) = 194 × [age]^−0.287^ × [serum creatinine (mg/dL)]^−1.094^ × 0.739. A 50 % decline in eGFR from baseline was detected at the first of three consecutive visits when the renal event occurred. Furthermore, the correlations of serum creatinine (Cre) concentrations measured at respective institutions and the central laboratories were examined, and the former concentrations corrected with the latter ones were used to address the interinstitutional heterogeneity of the data obtained. Furthermore, the functional relationship analysis estimating errors in the values measured at respective institutions and the central laboratories was conducted to make necessary corrections. The central laboratories measured serum Cre, urinary excretion of albumin (Alb), parathyroid hormone, and fibroblast growth factor (FGF) 23 concentration.

### Secondary endpoints

The following four renal events were established as the secondary endpoints: (1) rate of decline in eGFR from baseline. The slope of the regression line was calculated based on all eGFRs that had been determined between the onset of the study and the final determination point of eGFR and on the number of days from the onset of the study; (2) time to a 50 % decline in eGFR from baseline; (3) time to the initiation of RRT; and (4) time to doubling of serum Cre concentration. The number of days before the first of three consecutive visits after the onset of the study, at which serum Cre concentration doubled against the baseline value, was determined.

### Candidate risk factors for CKD progression to ESRD

Among traditional and nontraditional risk factors, we examined the following as candidate risk factors for CKD progression to ESRD that were assessed with the renal event-based primary and secondary endpoints: age, gender, complication of DM, history of CVD, body mass index (BMI), systolic blood pressure (SBP), diastolic blood pressure (DBP), concurrent medications [erythropoiesis-stimulating agents (ESAs), ARBs and/or ACEIs], hemoglobin (Hb), Alb, C-reactive protein, blood urea nitrogen (BUN), Cre, eGFR, and urine albumin-to-creatinine ratio (UACR).

## Associations of BPs with the primary endpoint

### Blood pressure pattern 1

Patients were categorized to two BP groups (the ≥140 mmHg in SBP and/or ≥90 mmHg in DBP group; and the <140 mmHg in SBP and <90 mmHg in DBP group), and these two groups were then compared with respect to the primary endpoint.

### Blood pressure pattern 2

Patients were categorized to the following seven BP groups according to the Japanese Society for Hypertension’s criteria: optimal (<120 mmHg in SBP and <80 mmHg in DBP), normal (120–129 mmHg in SBP and/or 80–84 mmHg in DBP), high normal (130–139 mmHg in SBP and/or 85–89 mmHg in DBP), grade 1 hypertension (140–159 mmHg in SBP and/or 90–99 mmHg in DBP), grade 2 hypertension (160–179 mmHg in SBP and/or 100–109 mmHg in DBP), grade 3 hypertension (≥180 mmHg in SBP and/or ≥110 mmHg in DBP), and isolated systolic hypertension (<140 mmHg in SBP and <90 mmHg in DBP), and seven groups were then compared with respect to the primary endpoint.

### Statistical analyses

The associations of risk factors with CKD progression to ESRD were analyzed according to the Cox and Poisson regression models, preceded by the confirmation of correlations among risk factors and of multicollinearity. Regression diagnosis using the lever and covariate ratios was made to assess the observed values with a great association. The log–log plots were prepared in Cox regression, while residual diagnosis was made in Poisson regression. The models considering interactions were applied to both Cox and Poisson regressions in an attempt to select risk factors according to the stepwise method. The multivariate Cox proportional hazards models were used to explore the risk factors for the primary and secondary endpoints mentioned above. We did not establish the threshold for correlation coefficient but verified that all variables used had a correlation coefficient of less than 0.6, thus showing no severe multicollinearity (variance inflation factor: <10). Multivariate analyses were made using the data from 1331 patients about whom all variables had been measured in the complete case analysis, and we used the multiple imputation method to handle the missing data.

The Kaplan–Meier curves were prepared to estimate the cumulative incidence of the primary endpoint, and the log-rank test was conducted to compare two and seven groups with respect to BP patterns 1 and 2, respectively. A two-tailed value of *P* < 0.05 was considered statistically significant. All statistical analyses were made using SAS version 9.4 (SAS Institute Inc., Cary, NC).

## Results

### Baseline characteristics of patients

**Fig. 1:**
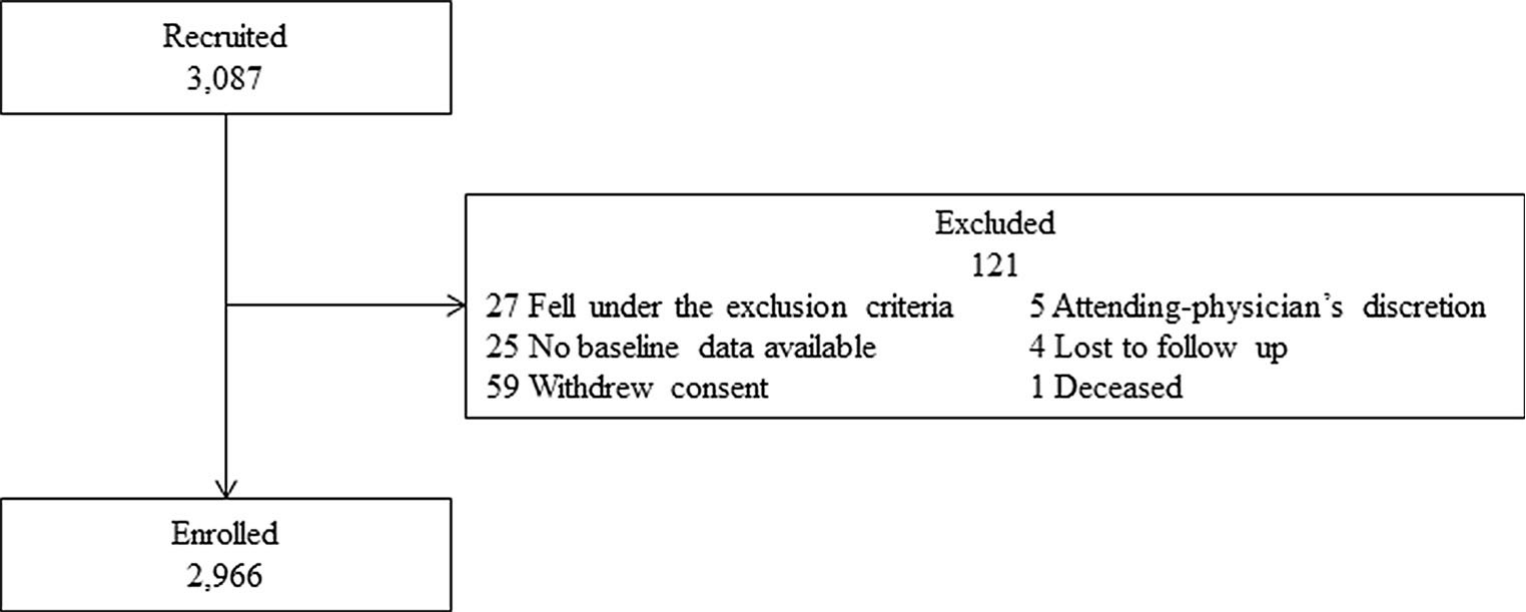
Patient disposition

A total of 2966 patients were enrolled (Fig. 1), and patient characteristics at baseline by CKD stage are shown in Table 1. Females accounted for 37.9 %, and the age of the patients was 60.3 ± 11.6 years (mean ± SD); 43.8 % of the patients were elderly (65–77 years of age). Furthermore, 37.7 and 31.6 % of the patients had DM and increased UACR ≥1000 mg/g Cre, respectively. The mean eGFR was 28.9 ± 12.2 mL/min/1.72 m^2^. The median duration of follow-up was approximately 3.9 years. In concert with CKD stage at baseline, the higher values (*P* < 0.0001) were obtained with respect to age, history of CVD, SBP, pulse pressure, BUN, uric acid (UA), UACR 300–999 mg/g Cre, UACR ≥1000 mg/g Cre, ESAs, and sodium bicarbonate, as well as serum Cre, phosphorus, parathyroid hormone, and log FGF23; the lower values (*P* < 0.0001) were obtained with respect to BMI, eGFR, and Hb, as well as serum Alb, calcium (Ca), and lipids.

**Table 1:** Patient characteristics at baseline by chronic kidney disease stage

Characteristics	(*n*)	Chronic kidney disease stages				Trend *P*
		Stage G3a, *n* (306)	Stage G3b, *n* (1045)	Stage G4, *n* (1149)	Stage G5, *n* (466)	
Age, years	60. 3 ± 11.6 (2966)	54.7 ± 13.4	59.9 ± 12.0	61.5 ± 10.6	62.1 ± 10.6	<0.0001
Female, %	37.9 (2966)	36.9	35.4	38.6	42.7	0.018
Diabetes mellitus, %	37.7 (2966)	31.4	36.3	38.8	42.1	0.001
History of cardiovascular disease, %	23.0 (2966)	16.7	21.3	24.0	28.5	<0.0001
Body mass index, kg/m^2^	23.5 ± 3.8 (2678)	24.3 ± 3.6	23.7 ± 3.9	23.3 ± 3.8	23.2 ± 3.7	<0.0001
Systolic blood pressure, mmHg	131.7 ± 18.6 (2923)	129.0 ± 17.3	130.1 ± 17.9	132.1 ± 19.1	136.2 ± 19.0	<0.0001
Diastolic blood pressure, mmHg	76.3 ± 11.8 (2920)	76.3 ± 11.3	76.5 ± 11.4	76.0 ± 12.2	76.4 ± 12.2	0.966
Pulse pressure, mmHg	55.4 ± 14.3 (2921)	52.7 ± 13.1	53.6 ± 13.8	56.1 ± 14.5	59.7 ± 14.3	<0.0001
Current smoker, %	44.9 (2521)	43.7	46.2	45.2	41.9	0.454
eGFR, mL/min/1.73 m^2^	28.9 ± 12.2 (2966)	50.5 ± 4.9	37.1 ± 4.2	22.5 ± 4.3	11.8 ± 2.0	<0.0001
Serum creatinine, mg/dL	2.15 ± 1.06 (2966)	1.11 ± 0.17	1.44 ± 0.25	2.29 ± 0.52	4.05 ± 0.90	<0.0001
Blood urea nitrogen, mg/dL	31.5 ± 14.7 (2925)	19.0 ± 5.0	23.5 ± 6.6	34.1 ± 11.5	50.9 ± 17.0	<0.0001
Hemoglobin, g/dL	12.1 ± 1.8 (2920)	13.3 ± 1.8	12.8 ± 1.8	11.7 ± 1.6	10.6 ± 1.3	<0.0001
Uric acid, mg/dL	7.18 ± 1.56 (2901)	6.59 ± 1.48	7.03 ± 1.39	7.35 ± 1.60	7.49 ± 1.73	<0.0001
Serum albumin, g/dL	3.97 ± 0.43 (2870)	4.09 ± 0.41	4.01 ± 0.42	3.93 ± 0.42	3.89 ± 0.45	<0.0001
Serum calcium, mg/dL	9.01 ± 0.53 (2718)	9.21 ± 0.43	9.11 ± 0.46	8.99 ± 0.49	8.70 ± 0.65	<0.0001
Serum phosphorus, mg/dL	3.53 ± 0.69 (2624)	3.27 ± 0.57	3.31 ± 0.59	3.55 ± 0.62	4.11 ± 0.77	<0.0001
Serum parathyroid hormone, pg/mL	106.3 ± 92.6 (2755)	56.0 ± 26.4	68.1 ± 34.9	109.2 ± 70.9	216.2 ± 147.2	<0.0001
Total cholesterol, mg/dL	194.2 ± 43.3 (2479)	201.6 ± 43.8	197.0 ± 43.8	192.1 ± 41.1	187.5 ± 45.8	<0.0001
High-density cholesterol, mg/dL	54.5 ± 18.4 (2126)	57.2 ± 18.5	56.0 ± 18.6	53.5 ± 18.3	51.5 ± 17.5	<0.0001
Low-density cholesterol, mg/dL	108.2 ± 33.0 (2231)	114.0 ± 28.6	110.1 ± 34.5	106.4 ± 31.0	103.8 ± 36.2	<0.0001
Triglycerides, mg/dL	167.0 ± 109.3 (2512)	175.2 ± 133.2	165.9 ± 108.4	168.6 ± 110.4	159.5 ± 87.2	0.097
Ferritin, ng/mL	137.2 ± 136.6 (1539)	130.4 ± 124.6	124.8 ± 120.5	142.6 ± 153.0	153.0 ± 131.5	0.041
C-reactive protein, mg/dL	0.10 (0.04–0.20)	0.08 (0.04–0.15]	0.10 (0.04–0.20]	0.10 (0.04–0.20]	0.08 (0.03–0.20]	0.064^†^
Fibroblast growth factor 23, pg/mL	56.70 (40.10–91.30)	39.20 (30.70–51.30)	46.60 (35.90–64.10)	62.45 (46.80–94.00)	109.60 (71.30–176.80)	NC
Log fibroblast growth factor 23^a^	4.24 ± 0.91 (2709)	3.85 ± 0.83	4.01 ± 0.80	4.30 ± 0.89	4.85 ± 0.93	<0.0001
UACR 300–999 mg/g Cre, %	28.2 (2713)	21.1	27.1	30.4	30.0	<0.0001
UACR ≥1000 mg/g Cre, %	31.6 (2713)	15.4	21.9	36.1	52.0	<0.0001
ARBs or ACEIs, %	81.8 (2966)	76.5	80.7	84.3	82.0	0.013
Erythropoiesis-stimulating agents, %	13.2 (2966)	1.0	2.9	15.5	38.6	<0.0001
Statins, %	40.3 (2966)	43.5	40.5	41.6	34.6	0.039
Vitamin D receptor activators, %	8.6 (2966)	9.2	7.2	7.6	13.7	0.009
Sodium bicarbonate, %	9.7 (2966)	1.0	3.7	11.0	25.8	<0.0001

Values are expressed as mean ± SD or median (interquartile range)

^†^Calculated based on mean ± SD

^a^Logarithmic conversion was conducted to allow inter-group comparisons

Stage G3a, eGFR: 45–59 mL/min/1.73 m^2^; stage G3b, eGFR: 30–44 mL/min/1.73 m^2^; stage G4, eGFR: 15–29 mL/min/1.73 m^2^; stage G5, eGFR: <15 mL/min/1.73 m^2^

*eGFR* estimated glomerular filtration rate, *NC* not calculable, *UACR* urine albumin-to-creatinine ratio, *ARBs* angiotensin receptor blockers, *ACEIs* angiotensin-converting enzyme inhibitors

### Time-course changes in the primary endpoint by CKD stage at baseline

**Fig. 2:**
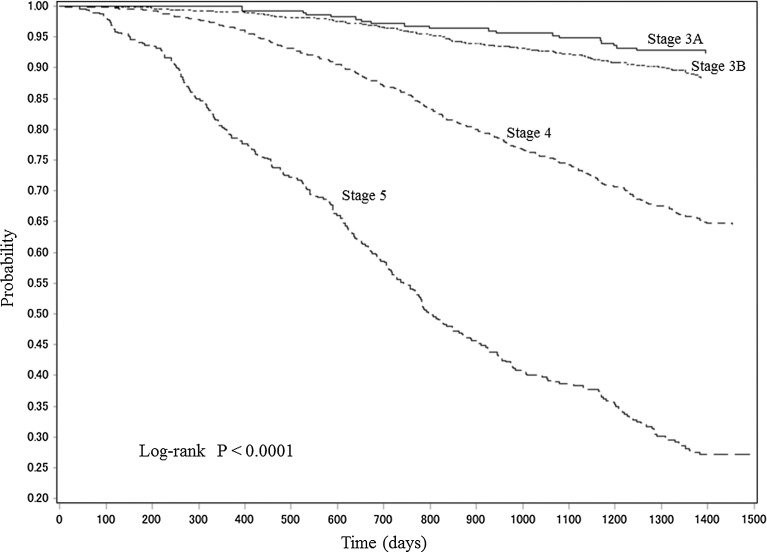
Kaplan–Meier curves for primary endpoint renal event-free Japanese patients with CKD by CKD stage at baseline. *CKD* chronic kidney disease

The Kaplan–Meier curves for primary endpoint renal event-free Japanese patients with CKD according to CKD stage at baseline are shown in Fig. 2. The incidence of the primary endpoint increased significantly (*P* < 0.0001) in concert with CKD stage at baseline.

### Associations of risk factors with the primary endpoint

Multivariate analysis disclosed that the following risk factors were significantly associated with the primary endpoint: current smoker, sodium bicarbonate, increases in BMI, SBP, UACR 300––999 mg/g Cre, UACR ≥1000 mg/g Cre, and serum Cre; and decreases in eGFR, serum Alb, and Hb. Among these variables, multivariate analysis revealed that only the following two variables showed a *P* value of <0.0001: elevated SBP at baseline [hazard ratio (HR) 1.203; 95 % confidence interval (CI) 1.099–1.318] and increased UACR at baseline (HR 4.523; 95 % CI 3.098–6.604) (Table 2).

**Table 2:** Associations of variables with a composite of time to a 50 % decline in estimated glomerular filtration rate from baseline or time to the initiation of renal replacement therapy of Japanese patients with chronic kidney disease according to the multiple Cox regression analysis

Variables	Univariate		Multivariate (*n* = 1331)	
	HR (95 % CI)	*P* value	HR (95 % CI)	*P* value
Age, per 1 year greater	1.001 (0.994–1.008)	0.767	0.988 (0.975–1.001)	0.068
Male gender	1.600 (1.344–1.904)	<0.0001	1.272 (0.884–1.831)	0.195
Diabetes mellitus	1.663 (1.418–1.949)	<0.0001	0.907 (0.694–1.185)	0.474
History of cardiovascular disease	1.295 (1.080–1.552)	0.005	0.823 (0.628–1.078)	0.158
Body mass index, per kg/m^2^ greater	1.029 (1.007–1.051)	0.011	1.050 (1.014–1.087)	0.006
Systolic blood pressure, per 10 mmHg greater	1.213 (1.165–1.264)	<0.0001	1.203 (1.099–1.318)	<0.0001
Diastolic blood pressure, per 10 mmHg greater	1.290 (0.700–2.379)	0.415	0.852 (0.368–1.975)	0.709
Current smoker^†^	1.730 (1.413–2.118)	<0.0001	1.542 (1.126–2.113)	0.007
Ex-smoker^a^	1.232 (1.023–1.483)	0.028	1.253 (0.931–1.686)	0.138
Estimated glomerular filtration rate	0.903 (0.895–0.912)	<0.0001	0.969 (0.944–0.995)	0.021
Uric acid	1.134 (1.078–1.192)	<0.0001	0.948 (0.882–1.019)	0.144
Serum albumin	0.317 (0.268–0.374)	<0.0001	0.731 (0.540–0.989)	0.042
Serum creatinine	2.455 (2.313–2.605)	<0.0001	1.589 (1.228–2.056)	<0.001
Blood urea nitrogen	1.044 (1.041–1.048)	<0.0001	0.997 (0.985–1.008)	0.585
Hemoglobin	0.707 (0.675–0.740)	<0.0001	0.858 (0.781–0.941)	0.001
Total cholesterol	0.999 (0.997–1.001)	0.325	1.000 (0.998–1.003)	0.734
High-sensitivity C-reactive protein	1.077 (0.977–1.187)	0.135	1.037 (0.941–1.142)	0.466
Serum phosphorus	1.945 (1.783–2.121)	<0.0001	1.207 (0.982–1.484)	0.074
Serum calcium	0.302 (0.266–0.343)	<0.0001	0.792 (0.604–1.038)	0.091
Log fibroblast growth factor 23	1.416 (1.324–1.514)	<0.0001	0.992 (0.853–1.153)	0.916
UACR, 300–999 mg/g Cre	3.805 (2.866–5.051)	<0.0001	2.127 (1.432–3.160)	<0.001
UACR, ≥1000 mg/g Cre	10.444 (8.049–13.552)	<0.0001	4.523 (3.098–6.604)	<0.0001
ARBs and/or ACEIs	1.361 (1.082–1.711)	0.008	0.848 (0.608–1.183)	0.332
Erythropoiesis-stimulating agents	2.931 (2.437–3.526)	<0.0001	0.908 (0.671–1.227)	0.529
Statins	1.050 (0.894–1.232)	0.552	0.931 (0.732–1.185)	0.563
Sodium bicarbonate	2.590 (2.114–3.174)	<0.0001	1.340 (1.004–1.788)	0.047

^a^Against the reference “nonsmoker”

*HR* hazard ratio, *CI* confidence interval, *UACR* urine albumin-to-creatinine ratio, *ARBs* angiotensin receptor blockers, *Cre* creatinine, *ACEIs* angiotensin-converting enzyme inhibitors

### Associations of risk factors with the secondary endpoints

1. Rates of decline in eGFR.
  The rates of decline in eGFR per year exhibited a significant tendency to be higher for greater CKD stage and higher UACR (trend *P* = 0.003 and <0.0001, respectively; Table 3). Especially, the mean of the rates in patients who had increased UACR decreased significantly (*P* < 0.0001) by eightfold faster than those who had a UACR of <300 mg/g Cre.

**Table 3:** Rates of decline in eGFR per year

	Mean ± SD	*P* value	Trend *P*
eGFR			
Stage G3a	−1.925 ± 5.681	0.003	0.003
Stage G3b	−2.056 ± 5.924		
Stage G4	−3.182 ± 14.189		
Stage G5	−3.754 ± 6.374		
Hypertension			
Group 1	−3.480 ± 6.650	0.154	
Group 2	−2.641 ± 10.440		
UACR (mg/g Cre)			
<300	−0.546 ± 4.690	<0.0001	<0.0001
300–999	−2.399 ± 6.297		
≥1000	−4.560 ± 4.894		

Values are expressed as mean ± SD

Stage G3a, eGFR: 45–59 mL/min/1.73 m^2^; stage G3b, eGFR: 30–44 mL/min/1.73 m^2^; stage G4, eGFR: 15–29 mL/min/1.73 m^2^; stage G5, eGFR: <15 mL/min/1.73 m^2^; Group 1, ≥140 mmHg in SBP and/or ≥90 mmHg in DBP; Group 2, <140 mmHg in SBP and <90 mmHg in DBP

*eGFR* estimated glomerular filtration rate, *UACR* urine albumin-to-creatinine ratio, *Cre* creatinine, *DBP* diastolic blood pressure, *SBP* systolic blood pressure
  Furthermore, multiple Cox regression analysis revealed that the following risk factors were significantly associated with the rate of decline in eGFR: younger age and male gender; increases in SBP, UACR 300–999 mg/g Cre, and UACR ≥1000 mg/g Cre, UA, BUN, and serum phosphorus; and decreases in BMI and serum Ca (Supplement 1).
2. Time to a 50 % decline in eGFR from baseline.
  Multiple Cox regression analysis revealed that the following risk factors were significantly associated with time to a 50 % decline in eGFR from baseline: male gender, and administration of ESAs; increases in BMI, SBP, serum phosphorus, 300–999 mg/g Cre, and ≥1000 mg/g Cre; and decreases in eGFR, UA, serum Alb, and Hb (Supplement 2).
3. Time to the initiation of RRT.
  Multiple Cox regression analysis revealed that the following risk factors were significantly associated with time to the initiation of RRT: younger age, male gender, history of CVD, and administration of sodium bicarbonate; increases in SBP, UACR 300–999 mg/g Cre, UCAR ≥1000 mg/g Cre, serum Cre, and log FGF23; and decreases in eGFR, serum Alb, and Hb (Supplement 3).
4. Time to doubling of serum Cre concentration.
  Multiple Cox regression analysis revealed that the following risk factors were significantly associated with time to doubling of serum Cre concentration: younger age, male gender, and administration of ESAs; increases in BMI, SBP, UACR 300–999 mg/g Cre, and UCAR ≥1000 mg/g Cre; and decreases in eGFR, UA, serum Alb, and Hb (Supplement 4).

### Incidence rates by CKD stage of the primary and secondary endpoints

The incidence rates (1000 person-years of follow-up) of both the primary and secondary endpoints increased in nearly full association with CKD stage at baseline (Table 4).

**Table 4:** Incidence rates of the primary and secondary endpoints

CKD stages	Primary endpoint^a^	Secondary endpoints			
		Rate of decline in eGFR from baseline	Time to a 50 % decline in eGFR from baseline	Time to the initiation of RRT	Time to doubling of serum Cre concentration
G3a	18.3	NC	17.4	4.5	16.4
G3b	28.1	NC	27.0	13.1	22.8
G4	96.4	NC	82.0	63.1	68.9
G5	439.4	NC	101.1	265.7	68.2

Values are expressed as number of renal events (rate per 1000 person-years of follow-up)

^a^A composite of time to a 50 % decline in eGFR from baseline or time to the initiation of RRT

Stage G3a, eGFR: 45–59 mL/min/1.73 m^2^; stage G3b, eGFR: 30–44 mL/min/1.73 m^2^; stage G4, eGFR: 15–29 mL/min/1.73 m^2^; stage G5, eGFR: <15 mL/min/1.73 m^2^

*CKD* chronic kidney disease, *eGFR* estimated glomerular filtration rate, *RRT* renal replacement therapy, *Cre* creatinine, *NC* not calculable

### Primary endpoint according to two BP patterns at baseline

**Fig. 3:**
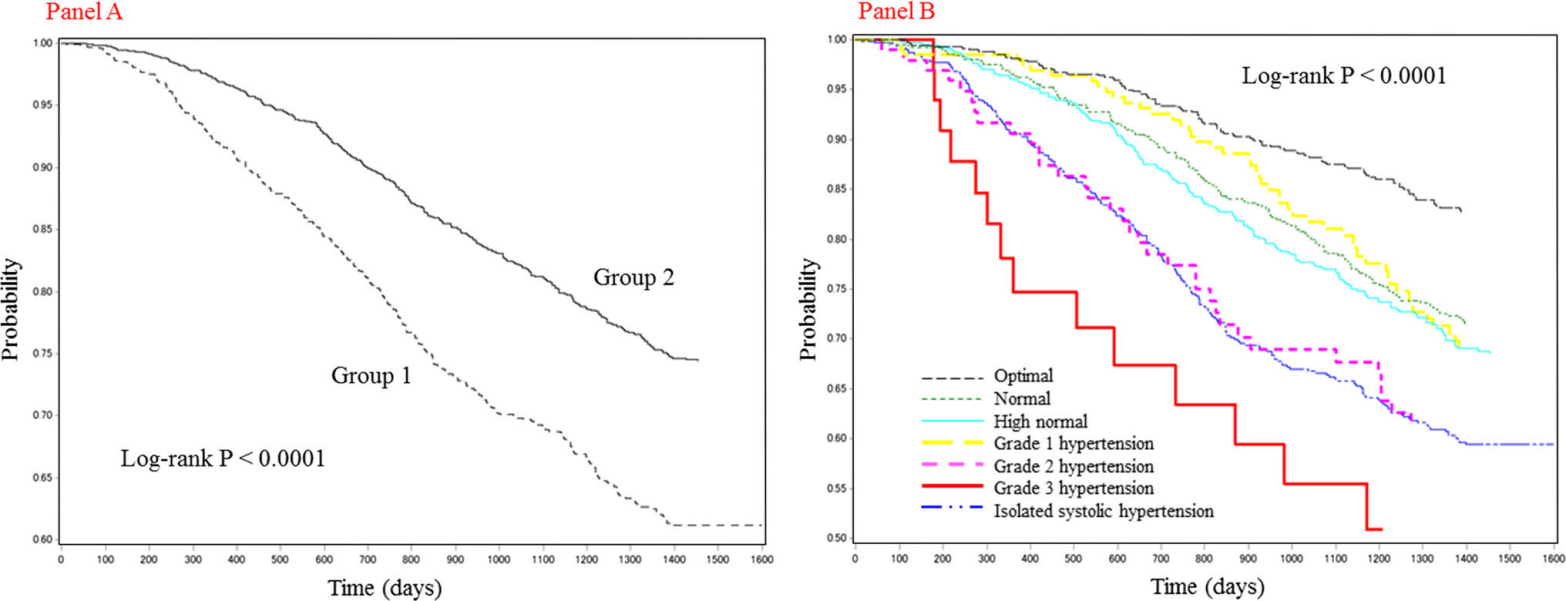
*Panel* **a** Kaplan–Meier curves for primary endpoint renal event-free Japanese patients with CKD in two blood pressure pattern groups formed according to pattern 1. Group 1, ≥140 mmHg in SBP and/or ≥90 mmHg in DBP; Group 2, <140 mmHg in SBP and <90 mmHg in DBP. *Panel* **b** Kaplan–Meier curves for primary endpoint renal event-free Japanese patients with CKD in seven blood pressure pattern groups formed according to pattern 2. *CKD* chronic kidney disease, *SBP* systolic blood pressure, *DBP* diastolic blood pressure

The incidences of the primary endpoint were compared between the following two BP pattern groups according to BP pattern 1: the ≥140 mmHg in SBP and/or ≥90 mmHg in DBP group; and the <140 mmHg in SBP and <90 mmHg in DBP group. The Kaplan–Meier curves for primary endpoint renal event-free Japanese patients with CKD according to BP pattern 1 are shown (Fig. 3a). The incidence of the primary endpoint increased significantly (*P* < 0.0001) in the ≥140 mmHg in SBP and/or ≥90 mmHg in DBP group. The incidences of the primary endpoint were compared among the following seven BP category groups according to BP pattern 2: the optimal group; the normal group; the high normal group; the grade 1 hypertension group; the grade 2 hypertension group; the grade 3 hypertension group; and the isolated systolic hypertension group. The Kaplan–Meier curves for primary endpoint renal event-free Japanese patients with CKD according to BP pattern 2 in seven groups are shown (Fig. 3b). A significant difference (*P* < 0.0001) was found among the seven groups with respect to the primary endpoint.

### Primary endpoint according to UACR at baseline

**Fig. 4:**
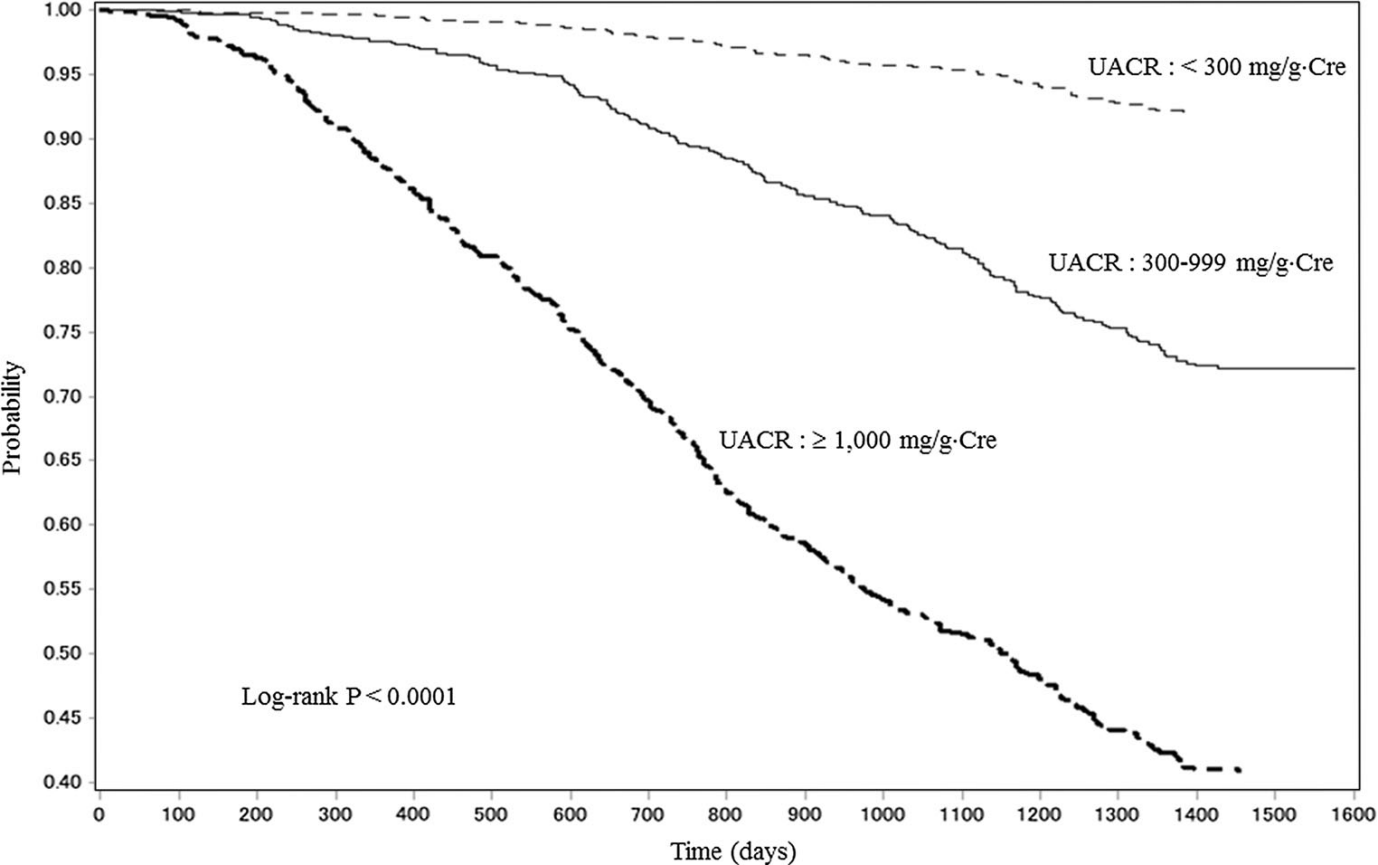
Kaplan–Meier curves for Japanese patients with CKD who developed renal events according to three categories (<300, 300–999, and ≥1000 mg/g Cre) of UACR. *CKD* chronic kidney disease, *Cre* creatinine, *UACR* urine albumin-to-creatinine ratio

The incidences of the primary endpoint were calculated in three UACR (<300, 300–999, and ≥1000 mg/g Cre) categories. The Kaplan–Meier curves for Japanese patients with CKD according to these three categories of UACR at baseline are shown in Fig. 4. The incidence of the primary endpoint increased significantly (*P* < 0.0001) among the three UACR groups.

## Discussion

The present study is the first, large-scale, multicenter, prospective cohort study in Japanese patients with CKD under nephrology care, which aimed at identifying risk factors for CKD progression to ESRD. Our study disclosed the following facts: (1) elevated SBP and increased UACR, both at baseline, were significantly associated (HR 1.203, 95 % CI 1.099–1.318 and HR 4523, 95 % CI 3.098–6.604; *P* < 0.0001, respectively) with the primary endpoint; and (2) the deterioration of a powerful predictor of ESRD, proteinuria (which was translated into higher UACR), accelerated CKD progression to ESRD extensively. These findings are in concert with previous clinical studies indicating that lower eGFR and higher UACR are independently associated with the increased risks of CVD and ESRD.

Numerous previous studies have indicated that hypertension is one of the important risk factors for renal impairment. In the present study, SBP at baseline was significantly associated with both the primary and secondary endpoints. On the other hand, DBP was not associated with a decline in renal function. Furthermore, the primary endpoint was analyzed according to two patterns of BPs. Consequently, the number of patients who developed a primary endpoint renal event increased in parallel with the severity of hypertension at baseline—a finding that is concordant with a previous study [9]. Statistical analyses according to two patterns of BPs revealed that patients with isolated systolic hypertension, which prevails in the elderly, showed no marked changes in the primary endpoint.

In the present study, increased UACR was significantly associated with the rate of decline in eGFR from baseline. In general, proteinuria, considered to reflect the severity of glomerular damage and to be a risk factor for systemic angiopathy, has also been reported to be a risk factor for renal impairment or CVD [10]. A massive health checkup program was conducted to annually follow up ≥120,000 Japanese individuals in the general population to examine time-course changes in eGFR for not less than 10 years [11]. The study showed an annual average decrease of 0.36 mL/min/1.73 m^2^ in eGFR and revealed that hypertension, proteinuria, and low eGFR at baseline accelerated the decline in the renal function of investigated patients. On the other hand, the results from the present study indicate that the rate of decline in eGFR from baseline in Japanese patients with CKD increased in parallel with CKD progression; the rate was >tenfold greater in patients with CKD stage G5. In the US, Yang et al. [12] conducted a large-scale, prospective cohort study (the CRIC study) in patients with CKD. Regarding ESRD and eGFR halving from baseline, the HRs in the highest proteinuria category compared to the lowest counterpart were 11.83 (95 % CI 8.40–16.65) and 11.19 (95 % CI 8.53–14.68), respectively. They considered that proteinuria was a strong risk factor for the latter two endpoints only. CKD differs between the US and Japan with respect to its background factors, e.g., the proportion of underlying disease, the complication rate of CAD, and BMI. Nevertheless, we also verified the association of higher UACR, an index for ESRD, with declined eGFR. We established three categories of UACR to examine Alb excretion in spot urine samples in reference to the guideline [7]. Consequently, multivariate analysis revealed that *P* < 0.0001 was calculated not for UACR >300 mg/g Cre, the guideline’s category A3, but for UACR ≥1000 mg/g Cre, an arbitrary category. This novel finding indicates that CKD patients with increased UACR are at greater risk of experiencing CKD progression to ESRD compared to those with UACR 300–999 mg/g Cre among those with a high UACR.

De Nicola et al. [13] examined the renal prognosis of patients with CKD stages G1–G4 who were under nephrology care. They used the composite outcome of ESRD or a ≥40 % decline in eGFR from baseline and established the therapeutic target values for BPs, anemia, and urine proteins to care for 729 patients. They found a significant association (HR 1.96) of DM with combined renal endpoints when using hypertensive nephropathy as reference. However, we did not find any strong association between concurrent DM and the primary endpoint in the present study. Not all of patients, who had DM as underlying disease, developed diabetic nephropathy; the proportion of the relevant patients was no more than 55 %, a figure that had been described in our prior study [2]. The present study seems to have included a number of diabetic patients whose underlying disease was nephrosclerosis, which probably led to failure in extracting DM as a risk factor. We speculate that this unexpected finding is presumably attributable to the fact that many patients with DM have hypertensive nephropathy concurrently.

Voormolen et al. [14] reported a significant association (HR 0.178) between serum phosphorus level and the rate of decline in eGFR from baseline in patients with stage 4/5 CKD and conjectured that the association was attributable to the progression of arteriosclerosis caused by protein and phosphorus loads. In the present study, however, we did not find any significant association of elevated serum phosphorus level with the primary or secondary endpoints. Furthermore, we detected significant associations (HRs 1.050–1.090) between elevated BMI and the primary endpoint and some secondary endpoints. Of significance was the fact that elevated BMI was extracted as a risk factor similar to a previous study in Japan [15] even in a cohort of patients with CKD whose mean BMI was as low as 23.5 kg/m^2^. Also, we consider that the present study is clinically relevant in that the primary and secondary endpoints were examined in patients with CKD who were under nephrology care; not less than 80 % of them received ARBs and/or ACEIs, and their BPs (132 ± 19 mmHg in SBP/76 ± 12 mmHg in DBP) were under control to the levels at which the target BPs were almost reached. Our study is distinguishably featured by the precise assessment of renal events, including the rate of decline in eGFR from baseline, through the more meticulous care (e.g., frequent blood collections) of patients as compared with previous studies in Westerners.

Our study has several limitations. First, patient characteristics were determined only at baseline and once. Second, the GFR to assess the renal function of patients was not precisely calculated based on inulin clearance but was estimated with the serum creatinine-based equation. Third, selection bias cannot be ruled out, because patients were mostly enrolled at large-sized hospitals that can provide nephrology care.

In conclusion, elevated SBP and increased UACR were the risk factors significantly associated with CKD progression to ESRD in Japanese patients with CKD. Therefore, clinicians should constantly give heed, in the routine clinical setting, especially to advanced CKD patients with poorly controlled hypertension and increased UACR in an attempt to curb CKD progression to ESRD.

## Electronic supplementary material

Below is the link to the electronic supplementary material.

Supplementary material 1 (DOCX 64 kb)

Supplementary material 2 (DOCX 62 kb)

Supplementary material 3 (DOCX 63 kb)

Supplementary material 4 (DOCX 63 kb)
