# Supplementary material for: Risk factors for CKD progression in Japanese patients: findings from the Chronic Kidney Disease Japan Cohort (CKD-JAC) study
Source: Clin Exp Nephrol. 2016 Jul 13;21(3):446–56. doi: 10.1007/s10157-016-1309-1 (PMC5486452; doi:10.1007/s10157-016-1309-1)
Supplement: Supplementary file 1 — Supplementary material 1 (DOCX 64 kb) [file 10157_2016_1309_MOESM1_ESM.docx]

Supplement 1 Associations of variables with the rate of decline in the estimated glomerular filtration rate from baseline in Japanese patients with chronic kidney disease: multivariate analysis

| Variables | n = 1,327 | |
| --- | --- | --- |
|  | Regression coefficient (95% CI) | P value |
| Age | 0.037 (0.012-0.062) | 0.004 |
| Male gender | -1.309 (-1.998 to -0.621) | < 0.001 |
| Diabetes mellitus | 0.067 (-0.449 to 0.583) | 0.800 |
| History of cardiovascular disease | 0.138 (-0.421 to 0.698) | 0.628 |
| Body mass index | -0.068 (-0.135 to -0.001) | 0.047 |
| Systolic blood pressure | -0.349 (-0.538 to -0.159) | < 0.001 |
| Diastolic blood pressure | -0.125 (-1.727 to 1.477) | 0.878 |
| Current smoker^†^ | 0.005 (-0.678 to 0.687) | 0.989 |
| Ex-smoker^†^ | 0.177 (-0.409 to 0.762) | 0.555 |
| Estimated glomerular filtration rate | 0.015 (-0.027 to 0.057) | 0.478 |
| Serum uric acid | 0.264 (0.109-0.418) | 0.001 |
| Serum albumin | 0.500 (-0.201 to 1.201) | 0.162 |
| Serum creatinine | -0.149 (-0.695 to 0.397) | 0.592 |
| Blood urea nitrogen | 0.027 (0.001 to 0.052) | 0.039 |
| Hemoglobin | 0.160 (-0.015 to 0.335) | 0.072 |
| Total cholesterol | -0.004 (-0.010 to 0.002) | 0.184 |
| C-reactive protein | -0.084 (-0.395 to 0.227) | 0.597 |
| Serum phosphorus | -0.607 (-1.043 to -0.171) | 0.006 |
| Serum calcium | 1.148 (0.571 to 1.725) | < 0.0001 |
| Log fibroblast growth factor 23 | -0.012 (-0.300 to 0.275) | 0.934 |
| UACR, 300-999 mg/g⋅Cre | -0.735 (-1.323 to -0.148) | 0.014 |
| UACR, ≥ 1,000 mg/g⋅Cre | -2.291 (-2.942 to -1.641) | < 0.0001 |
| ARBs or ACEIs | 0.226 (-0.402 to 0.854) | 0.481 |
| Erythropoiesis-stimulating agents | 0.438 (-0.308 to 1.185) | 0.250 |
| Statins | -0.396 (-0.874 to 0.082) | 0.104 |
| Sodium bicarbonate | -0.456 (-1.216 to 0.304) | 0.240 |

^†^: Against the reference “nonsmoker”

UACR, urine albumin-to-creatinine ratio; ARBs, angiotensin receptor blockers; ACEIs,

angiotensin-converting enzyme inhibitors
