# Supplementary material for: Risk factors for CKD progression in Japanese patients: findings from the Chronic Kidney Disease Japan Cohort (CKD-JAC) study
Source: Clin Exp Nephrol. 2016 Jul 13;21(3):446–56. doi: 10.1007/s10157-016-1309-1 (PMC5486452; doi:10.1007/s10157-016-1309-1)
Supplement: Supplementary file 2 — Supplementary material 2 (DOCX 62 kb) [file 10157_2016_1309_MOESM2_ESM.docx]

Supplement 2 Associations of variables with time to a 50% decline in the estimated glomerular filtration rate in Japanese patients with chronic kidney disease: multivariate analysis

| Variables | n = 1,331 | |
| --- | --- | --- |
|  | HR (95% CI) | P value |
| Age, per 1 year greater | 0.987 (0.973-1.001) | 0.064 |
| Male gender | 1.929 (1.327-2.804) | 0.001 |
| Diabetes mellitus | 0.966 (0.711-1.310) | 0.822 |
| History of cardiovascular disease | 0.864 (0.626-1.192) | 0.373 |
| Body mass index | 1.077 (1.036-1.120) | < 0.001 |
| Systolic blood pressure, per 10 mmHg greater | 1.157 (1.040-1.288) | 0.007 |
| Diastolic blood pressure, per 10 mmHg greater | 1.368 (0.505-3.702) | 0.538 |
| Current smoker^†^ | 1.326 (0.915-1.921) | 0.136 |
| Ex-smoker^†^ | 1.235 (0.883-1.726) | 0.218 |
| Estimated glomerular filtration rate | 0.950 (0.933-0.967) | < 0.0001 |
| Uric acid | 0.897 (0.823-0.978) | 0.013 |
| Serum albumin | 0.628 (0.434-0.908) | 0.014 |
| Blood urea nitrogen | 0.993 (0.980-1.007) | 0.328 |
| Hemoglobin | 0.828 (0.745-0.921) | 0.001 |
| Total cholesterol | 1.002 (0.998-1.005) | 0.361 |
| C-reactive protein | 1.059 (0.934-1.200) | 0.373 |
| Serum phosphorus | 1.239 (0.963-1.595) | 0.096 |
| Serum calcium | 0.911 (0.653-1.270) | 0.582 |
| Log fibroblast growth factor 23 | 0.952 (0.794-1.140) | 0.591 |
| UACR, 300-999 mg/g⋅Cre | 3.280 (1.991-5.404) | < 0.0001 |
| UACR, ≥ 1,000 mg/g⋅Cre | 6.757 (4.154-10.992) | < 0.0001 |
| ARBs or ACEIs | 0.764 (0.525-1.113) | 0.161 |
| Erythropoiesis-stimulating agents | 0.651 (0.433-0.978) | 0.039 |
| Statins | 0.882 (0.669-1.162) | 0.370 |
| Sodium bicarbonate | 1.114 (0.764-1.626) | 0.575 |

^†^: Against the reference “nonsmoker”

UACR, urine albumin-to-creatinine ratio; ARBs, angiotensin receptor blockers; ACEIs,

angiotensin-converting enzyme inhibitors
