# Supplementary material for: Risk factors for CKD progression in Japanese patients: findings from the Chronic Kidney Disease Japan Cohort (CKD-JAC) study
Source: Clin Exp Nephrol. 2016 Jul 13;21(3):446–56. doi: 10.1007/s10157-016-1309-1 (PMC5486452; doi:10.1007/s10157-016-1309-1)
Supplement: Supplementary file 3 — Supplementary material 3 (DOCX 63 kb) [file 10157_2016_1309_MOESM3_ESM.docx]

Supplement 3 Associations of variables with time to the initiation of renal replacement therapy in Japanese patients with chronic kidney disease: multivariate analysis

| Variables | n = 1,331 | |
| --- | --- | --- |
|  | HR (95% CI) | P value |
| Age, per 1 year greater | 0.982 (0.968-0.996) | 0.011 |
| Male gender | 1.679 (1.091-2.583) | 0.018 |
| Diabetes mellitus | 0.892 (0.657-1.210) | 0.461 |
| History of cardiovascular disease | 0.684 (0.501-0.935) | 0.017 |
| Body mass index | 1.033 (0.994-1.074) | 0.102 |
| Systolic blood pressure, per 10 mmHg greater | 1.291 (1.165-1.432) | < 0.0001 |
| Diastolic blood pressure, per 10 mmHg greater | 0.826 (0.332-2.058) | 0.682 |
| Current smoker^†^ | 1.347 (0.930-1.951) | 0.115 |
| Ex-smoker^†^ | 1.307 (0.945-1.808) | 0.106 |
| Estimated glomerular filtration rate | 0.933 (0.897-0.971) | 0.001 |
| Uric acid | 0.968 (0.895-1.048) | 0.425 |
| Serum albumin | 0.640 (0.456-0.900) | 0.010 |
| Serum creatinine | 1.530 (1.138- 2.058) | 0.005 |
| Blood urea nitrogen | 1.002 (0.991-1.014) | 0.678 |
| Hemoglobin | 0.835 (0.748-0.931) | 0.001 |
| Total cholesterol | 0.999 (0.996-1.002) | 0.528 |
| C-reactive protein | 0.892 (0.747-1.065) | 0.208 |
| Serum phosphorus | 1.181 (0.939-1.486) | 0.156 |
| Serum calcium | 0.812 (0.602-1.097) | 0.175 |
| Log fibroblast growth factor 23 | 1.191 (1.017-1.396) | 0.031 |
| UACR, 300-999 mg/g⋅Cre | 1.926 (1.219-3.042) | 0.005 |
| UACR, ≥ 1,000 mg/g⋅Cre | 3.752 (2.420-5.818) | < 0.0001 |
| ARBs or ACEIs | 0.716 (0.494-1.038) | 0.078 |
| Erythropoiesis-stimulating agents | 1.029 (0.752-1.408) | 0.860 |
| Statins | 1.088 (0.827-1.432) | 0.545 |
| Sodium bicarbonate | 1.431 (1.051-1.949) | 0.023 |

^†^: Against the reference “nonsmoker”

HR, hazard ratio; CI, confidence interval; UACR, urine albumin-to-creatinine ratio;

ARBs, angiotensin receptor blockers; ACEIs, angiotensin-converting enzyme inhibitors
