# Supplementary material for: Risk factors for CKD progression in Japanese patients: findings from the Chronic Kidney Disease Japan Cohort (CKD-JAC) study
Source: Clin Exp Nephrol. 2016 Jul 13;21(3):446–56. doi: 10.1007/s10157-016-1309-1 (PMC5486452; doi:10.1007/s10157-016-1309-1)
Supplement: Supplementary file 4 — Supplementary material 4 (DOCX 63 kb) [file 10157_2016_1309_MOESM4_ESM.docx]

Supplement 4 Associations of variables with time to doubling of serum creatinine concentration in Japanese patients with chronic kidney disease: multivariate analysis

| Variables | n = 1,331 | |
| --- | --- | --- |
|  | HR (95% CI) | P value |
| Age, per 1 year greater | 0.980 (0.965-0.995) | 0.008 |
| Male gender | 1.907 (1.262-2.881) | 0.002 |
| Diabetes mellitus | 0.877 (0.625-1.230) | 0.446 |
| History of cardiovascular disease | 0.919 (0.645-1.311) | 0.642 |
| Body mass index | 1.090 (1.045-1.137) | < 0.0001 |
| Systolic blood pressure, per 10 mmHg greater | 1.182 (1.052-1.328) | 0.005 |
| Diastolic blood pressure, per 10 mmHg greater | 1.309 (0.442-3.880) | 0.627 |
| Current smoker^†^ | 1.484 (0.991-2.222) | 0.055 |
| Ex-smoker^†^ | 1.423 (0.982-2.062) | 0.062 |
| Estimated glomerular filtration rate | 0.951 (0.933-0.969) | < 0.0001 |
| Uric acid | 0.863 (0.784-0.950) | 0.003 |
| Serum albumin | 0.557 (0.369-0.840) | 0.005 |
| Blood urea nitrogen | 0.996 (0.981-1.011) | 0.591 |
| Hemoglobin | 0.791 (0.703-0.889) | < 0.0001 |
| Total cholesterol | 1.001 (0.998-1.005) | 0.436 |
| C-reactive protein | 1.092 (0.961-1.241) | 0.178 |
| Serum phosphorus | 1.183 (0.894-1.564) | 0.239 |
| Serum calcium | 0.939 (0.649-1.359) | 0.740 |
| Log fibroblast growth factor 23 | 0.862 (0.696-1.068) | 0.174 |
| UACR, 300-999 mg/g⋅Cre | 3.443 (1.958-6.053) | < 0.0001 |
| UACR, ≥ 1,000 mg/g⋅Cre | 7.263 (4.189-12.594) | < 0.0001 |
| ARBs or ACEIs | 0.827 (0.542-1.263) | 0.379 |
| Erythropoiesis-stimulating agents | 0.504 (0.312-0.815) | 0.005 |
| Statins | 0.877 (0.649-1.185) | 0.392 |
| Sodium bicarbonate | 1.068 (0.696-1.638) | 0.763 |

^†^: Against the reference “nonsmoker”

HR, hazard ratio; CI, confidence interval; Cre, creatinine; UACR, urine albumin-to-creatinine ratio;

ARBs, angiotensin receptor blockers; ACEIs, angiotensin-converting enzyme inhibitors
